# Multi-scale cellular engineering: From molecules to organ-on-a-chip

**DOI:** 10.1063/1.5129788

**Published:** 2020-03-03

**Authors:** Ngan F. Huang, Ovijit Chaudhuri, Patrick Cahan, Aijun Wang, Adam J. Engler, Yingxiao Wang, Sanjay Kumar, Ali Khademhosseini, Song Li

**Affiliations:** 1 Department of Cardiothoracic Surgery, Stanford University, Stanford, California 94305, USA; 2 Stanford Cardiovascular Institute, Stanford University, Stanford, California 94305, USA; 3 Department of Mechanical Engineering, Stanford University, Stanford, California 94305, USA; 4 Department of Biomedical Engineering, Institute for Cell Engineering, Johns Hopkins University School of Medicine, Baltimore, Maryland 21205, USA; 5 Department of Surgery, School of Medicine, University of California Davis, Sacramento, California 95817, USA; 6 Department of Biomedical Engineering, University of California Davis, Davis, California 95616, USA; 7 Institute for Pediatric Regenerative Medicine, Shriners Hospitals for Children, Sacramento, California 95817, USA; 8 Department of Bioengineering, Jacob School of Engineering, University of California San Diego, La Jolla, California 92093, USA; 9 Department of Bioengineering, University of California Berkeley, Berkeley, California 94720, USA; 10 Department of Chemical and Biomolecular Engineering, University of California Berkeley, Berkeley, California 94720, USA; 11 Department of Bioengineering, University of California, Los Angeles, California 90095, USA; 12 Department of Radiological Sciences, University of California, Los Angeles, California 90095, USA; 13 California Nanosystems Institute, University of California, Los Angeles, California 90095, USA

## Abstract

Recent technological advances in cellular and molecular engineering have provided new insights into biology and enabled the design, manufacturing, and manipulation of complex living systems. Here, we summarize the state of advances at the molecular, cellular, and multi-cellular levels using experimental and computational tools. The areas of focus include intrinsically disordered proteins, synthetic proteins, spatiotemporally dynamic extracellular matrices, organ-on-a-chip approaches, and computational modeling, which all have tremendous potential for advancing fundamental and translational science. Perspectives on the current limitations and future directions are also described, with the goal of stimulating interest to overcome these hurdles using multi-disciplinary approaches.

## I. INTRODUCTION

**FIG. 1.**
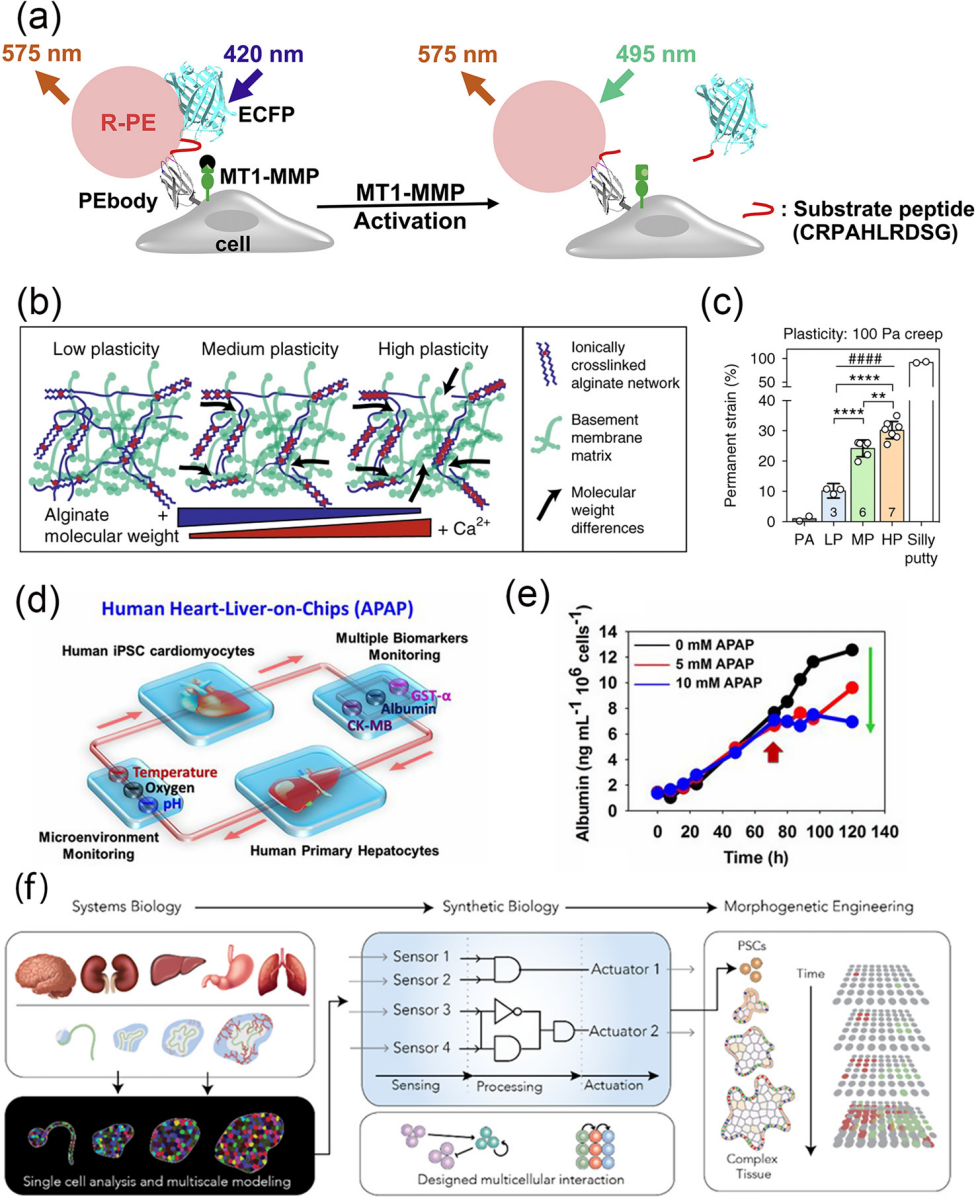
Examples of engineering strategies in molecular, extracellular, and microphysiological systems. (a) Molecular engineering of a biosensor for membrane-type-1 matrix metalloproteinase (MT1-MMP) activity based on changes in fluorescence emission. R-phycoerythrin (R-PE) fluorescence labeling of the intact biosensor allows energy transfer from enhanced cyan fluorescent protein (ECFP) to R-PE. When activated, MT1-MMP cleaves the biosensor substrate sequence, thereby disrupting fluorescence resonance energy transfer (FRET) and reducing the FRET/R-PE ratio. Reproduced with permission from Limsakul *et al.*, Cell Chem. Biol. **25**, 37 (2018). Copyright 2018 Elsevier.^7^ (b) Schematic of the approach to tuning matrix plasticity in interpenetrating networks (IPNs) of alginate (blue) and reconstituted basement membrane matrix (green) by varying the molecular weight of the alginate and ionic cross-linking. (c) By modulating the alginate molecular weight and degree of cross-linking, the permanent strain can be varied between low plasticity (LP), medium plasticity (MP), and high plasticity (HP) IPNs. Permanent strain, which was measured by creep-recovery tests, was significantly higher in HP IPNs, compared to MP and LP IPNs. For comparison, the permanent strain of polyacrylamide gels (PA) and silly putty are also provided. Statistically significant differences are indicated [**P < 0.01, ****P < 0.0001, analysis of variance (ANOVA)] and plasticity across the IPNs (####P < 0.0001, Spearman's rank correlation). Reproduced with permission from Wisdom *et al.*, Nat. Commun. **9**, 4144 (2018). Copyright 2018 Authors, licensed under a CC BY 4.0 (https://creativecommons.org/licenses/by/4.0/).^28^ (d) An example of a human heart-liver-on-a-chip for studying acetaminophen (APAP)-induced toxicity. Primary human hepatocytes and induced pluripotent stem cell (iPSC)-derived cardiomyocytes were linked together in a dual-organoid system, and APAP was then introduced into the chip for 72 h. (e) Using an electrode-based biosensor, albumin from hepatocytes could be quantified in the presence of APAP. The results show that albumin levels decreased in the presence of APAP, which is consistent with toxicity induced hepatic impairment. The arrow depicts the time when APAP was introduced. Reproduced with permission from Zhang *et al.*, Proc. Natl. Acad. Sci. U. S. A. **114**, E2293 (2017). Copyright 2017 National Academy of Science.^54^ (f) Schematic diagram depicts integrating systems and synthetic biology for morphogenetic engineering. Systems biology applied to development can generate circuits for engineering cell-intrinsic and cell–cell interactions that can be used to engineer complex, multi-cellular behaviors such as morphogenesis from pluripotent stem cells (PSCs). Reproduced with permission from Velazquez *et al.*, Trends Biotechnol. **36**, 415 (2018). Copyright 2018 Elsevier.^2^

Tissue and organ functions are largely dictated by complex molecular and cellular interactions. Such interactions contribute to homeostasis under physiological conditions and pathological disease progression. In the advent of innovative technologies in cellular and molecular bioengineering, the complex biological processes within tissues and organs are being elucidated at greater resolution than ever (Fig. 1). In addition, new insights and novel tools allow us to design and reconstitute complex living systems. At the molecular level, intrinsically disordered proteins (IDPs) and synthetic molecular probes enable the understanding and detection of molecular assemblies and subcellular structures, as well as functional assessment.^1^ At the single-cell and multi-cellular levels, inter-cellular communication and the integration of chemical, physical, and biological cues derived from the extracellular matrix (ECM) in a temporally and spatially resolved manner become increasingly important. The biophysical properties of the ECM, which modulate cellular behavior, include but are not limited to stiffness, viscoelasticity, and viscoplasticity, along with porosity, ligand patterning, spatial gradients, and three-dimensional (3D) structures in nano-, micro-, and macro-scales. The insights gained from molecular and cellular responses can be applied toward organ-on-a-chip approaches to better understand tissue morphogenesis, pathology, and cross talk between tissues and organs in integrated systems. Finally, with recent advances in computational modeling and bioinformatics, emerging multi-scale platforms that incorporate intra-cellular regulatory networks and inter-cellular interactions can be used to model complex multi-cellular processes.^2^ Here, we overview the latest advances and future directions in bioengineering at the molecular, cellular, and multi-cellular levels. As cellular and molecular bioengineering becomes increasingly more advanced, it is hoped that the insights gained and technologies developed can have a transformative impact in the fields of regenerative medicine, disease modeling, and development. This perspective is a product of the discussions at the 2019 Cell and Molecular Bioengineering Conference in Coronado, CA, USA, which highlights the breakthroughs and challenges in engineering biological complexity across length scales from macromolecules to cells and tissues.

## II. MOLECULAR SENSING AND CELLULAR SIGNALING

Molecular engineering has been widely explored as a robust approach to generate molecular sensors to dissect cell signaling and synthetic molecules for the assembly of multi-cellular structures and smart materials. We will focus on molecular sensing and signaling here and discuss extracellular molecular engineering in the later sections.

### A. IDPs and molecular engineering

Recent developments in molecular engineering strategies have provided new insights into molecular mechanisms of cellular functions. An emerging area of research is IDPs, which are proteins with extensively disorganized protein structures.^1,3^ IDPs have been shown to modulate phase transitions, leading to the condensation of nuclear bodies and organelles that modulate cellular processes.^1^ The development of light-controllable droplet assemblies based on phase transition can reveal molecular insights connecting biophysical properties and functional outcomes of molecular assemblies.^4,5^ Additionally, highly sensitive and specific biosensors based on fluorescence resonance energy transfer (FRET) and other signaling molecules are capable of visualizing the effects of IDPs on force generation across specific proteins such as focal adhesions in living cells. Future directions include simultaneous monitoring of multiple signaling molecules in living cells, the combination of signal sensing with functional actuation controls, and the development of non-invasive biophysical control using optical, electrical, and/or ultrasound technologies.

The integration of multi-scale computation and biophysical experiments is primed to reveal the key factors that determine the phase transition of IDPs.^6^ In the future, increasingly powerful computational algorithms and methods will become available to predict protein structures. Due to the plastic nature of IDP structures, traditional molecular dynamics simulation and homology modeling are limited in providing precise predictions of IDP conformations. The development of deep learning and machine learning algorithms, as well as artificial neural networks, should have significant impact under different physiological conditions. In conjunction with high-throughput screening approaches to integrate genetic library construction and deep sequencing technologies, it will become readily feasible to scan and characterize a large number of protein mutants experimentally in a relatively fast fashion. The iterative cross-comparison and adaptation between the computational and experimental results and strategies should lead to revolutionary progress in engineering new synthetic proteins, e.g., IDPs, and applying them to the imaging and controllable reprogramming of cellular functions.

### B. Synthetic protein engineering

The engineering of synthetic proteins, domains, and peptides is increasingly needed for various biological and biomedical applications. These engineered proteins can be used to study protein–protein interactions and to develop biosensors for cellular imaging. For example, directed evolution and high-throughput screening approaches have been integrated to develop a monobody variant (PEbody) capable of recognizing R-phycoerythrin (R-PE) that is fluorescent. Combined with another fluorescent protein, this engineered PEbody with R-PE can allow the tracking and visualizing of membrane bound matrix metalloproteinase (MMP) in living cells [Fig. 1(a)].^7^ Single chain antibodies (scFv), nanobodies, as well as other binding motifs, can also be similarly developed for imaging. Protein engineering can further be applied to develop therapeutic reagents. Indeed, numerous antibodies and their derivatives have been engineered for therapeutic purposes. For example, antibodies engineered with high specificity against checkpoint inhibitory pathways of the T-cell protein, PD1, and cytotoxic T-lymphocyte associated protein-4 (CTLA4) have led to revolutionary progress in cancer immunotherapy.^8^ Cytokines have also been reengineered to enhance efficacy while minimizing non-specific toxicity.^9^ The rational design of synthetic proteins requires the understanding of the molecular structure-function relationship and advanced computational simulation, and the selection and screening of designed proteins will rely on high-throughput *in vitro* cellular or multi-cellular systems.

**FIG. 2.**
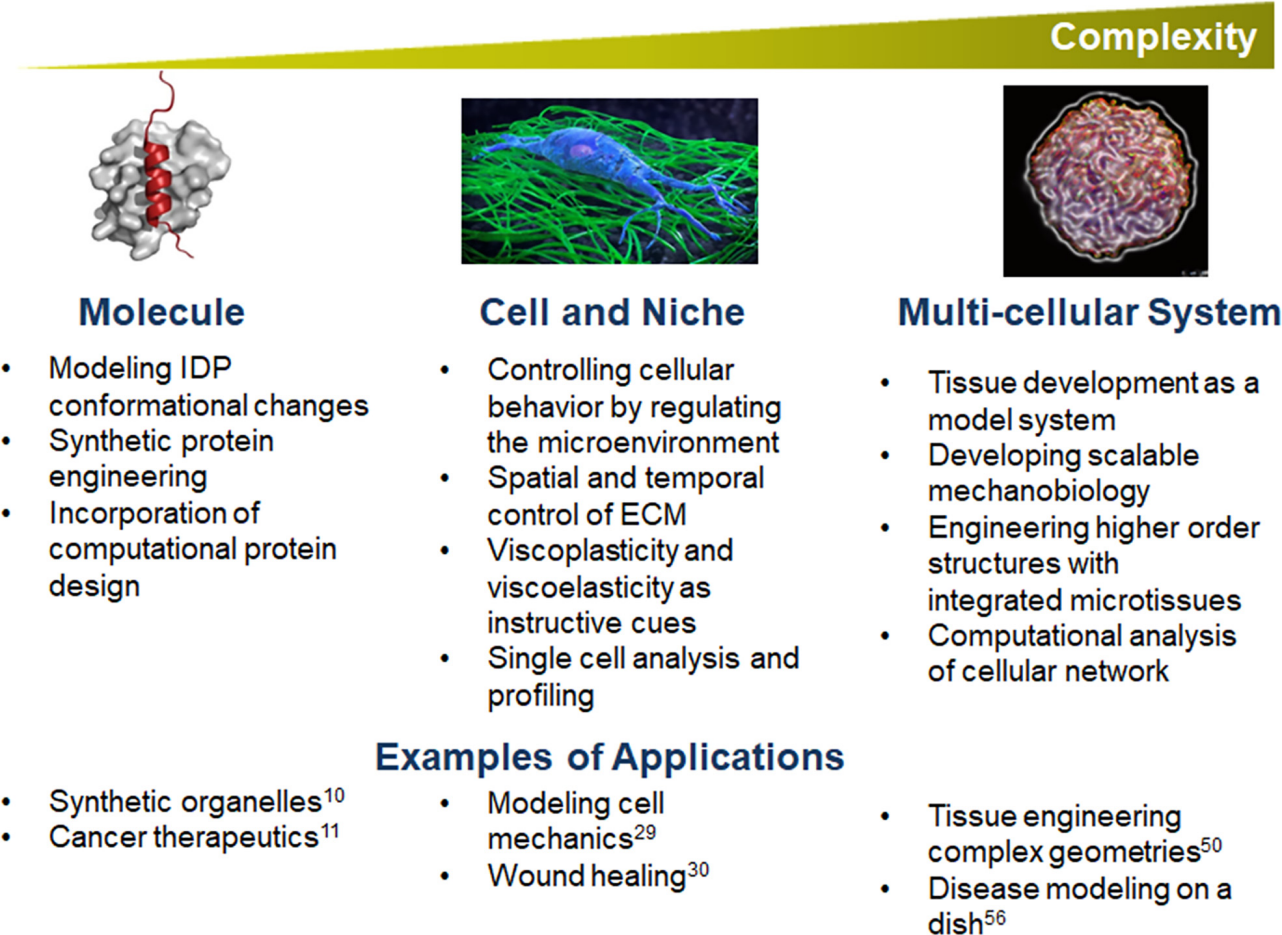
Current and emerging areas of research in engineering at the molecular, cellular, and multi-cellular levels. At the molecular level, IDP conformational changes and synthetic protein engineering can be applied toward the engineering of synthetic organelles and cancer therapeutics. At the cell-matrix level, innovations in spatiotemporal and mechanical tuning of the ECM enable more accurate modeling of cell mechanics and tissue dynamics like wound healing. At the multi-cellular level, scalable mechanobiology and higher order structures enable tissue engineering with increasing complexity and can be applied toward disease modeling. Reproduced with permission from Milles *et al.*, Prog. Nucl. Magn. Reson. Spectrosc. **109**, 79 (2018). Copyright 2018 Elsevier.^3^

With the rapid development of methods fostering library construction, high-throughput screening, and directed evolution strategies, overwhelmingly large numbers of different proteins/peptides can be engineered. These protein/peptides can be applied toward emerging areas such as engineering of synthetic organelles and cancer therapeutics (Fig. 2).^10,11^ For experiment-based protein engineering, a key remaining challenge is the efficient screening assay for desired functions. Recent advances in computational analysis and algorithms have made feasible the computational design of proteins. Based on the principle of protein folding at the lowest free energy state,^12^ computational algorithms and strategies have been successfully developed to find an amino acid sequence capable of folding into a desired structure. It is anticipated that the experimental assays based on directed evolution and computational methods will increasingly converge for integrative and novel approaches to allow the development of new generations of proteins/peptides for fundamental research and for diagnostic and therapeutic applications.

## III. ENGINEERING THE NICHE: MOVING FROM SINGLE CELLS TO MULTI-CELLULAR SYSTEMS

To move from a focus on molecular interactions within single cells to a focus on multi-cellular structures-on-a-chip with the capacity to function collectively as pseudo-organs, it is important to consider both extrinsic inter-cellular interactions and the extracellular niche. While the former may be self-explanatory or covered extensively elsewhere,^13^ the latter offers significant mechanobiological opportunities to control the multi-cellular behavior. The past decade provided numerous systems with exquisite control over this niche in a variety of contexts, but this originates with the observation that even the basic cellular building blocks of a tissue rely on the topography,^14^ stiffness,^15^ porosity,^16^ degradability,^17^ and composition^18^ of ECM to dictate behavior. In this section, we offer forward-looking observations of how next generation materials should control cells and multi-cellular structures. Namely, these include creating niches with spatial and temporal control of ECM properties to guide the scale-up from cells to organoids.

### A. Next generation materials: Control in time and space

While seminal observations a decade ago with individual cells on gels created a paradigm shift that resulted in the creation of mechanobiology as a field, so too will the next decade bring with it a series of new mechanobiological observations with multi-cellular structures and organoids. Cell–cell interactions are clearly important as stated above, but we argue that the biggest opportunity in the next decade for this field will be the development of increasingly dynamic engineered systems to improve our control over organoid systems. While not routine yet, leading work has shown that stem^19^ and cancer cells^20^ can show “memory” of their former niche as their ECM softens or stiffens; reversible topography shows equally dynamic responses in adult stem cells.^21^ Spatial changes can also play critical roles, regardless of the specific matrix properties, using newer techniques beyond conventional microcontact printing and soft lithography methods. For example, spatial gradients of stiffness, porosity, or ligand have become more common. We believe that the next decade will include significant growth in complex systems using multiple orthogonal patterns within a specific cue or single patterns of multiple cues.^22^ Together these approaches may pose a more realistic niche for questions of dynamic tissue-level behaviors associated with disease modeling and development.

### B. Beyond elasticity: Viscoelasticity and viscoplasticity

Although the role of matrix stiffness, or elasticity, in regulating cell behaviors is now increasingly well-understood, recent work has revealed the additional impact of matrix viscoelasticity and viscoplasticity in regulating cell behaviors. Many soft tissues and extracellular matrices are viscoelastic, exhibiting stress relaxation in response to a deformation, creep in response to a mechanical stress, or dissipating mechanical energy imparted into the material.^23,24^ Sources of viscoelasticity include the unbinding of weak, non-covalent bonds that link the matrix components together and the dissipation of energy that accompanies the movement of fluid through the matrix. Utilizing substrates with tunable viscoelastic properties, recent studies have revealed that the time-dependent relaxation or creep properties of the matrix impact cell spreading, proliferation, matrix formation, and stem cell differentiation in both two-dimensional (2D) and 3D culture systems.^24–27^ Mechanistic studies indicate that matrix viscoelasticity is sensed by cells through integrin clustering, cytoskeletal tension, and, in 3D culture, gauging of resistance to cell volume expansion. Many viscoelastic matrices can also exhibit mechanical plasticity or irreversible deformations in response to a mechanical stress or strain. For example, interpenetrating networks (IPNs) of alginate and the reconstituted basement membrane matrix with varying molecular weights result in a range in permanent strain [Figs. 1(b) and 1(c)].^28^ Matrix mechanical plasticity has been recently found to be a key regulator of regulate cell migration, with cancer cells found to migrate through nanoporous matrices, independent of proteases when the ECM exhibits sufficient matrix mechanical plasticity.^28^ Thus, matrix viscoplasticity may be related to the idea of confinement, with increased viscoplasticity corresponding to decreased confinement.

Given that the role of matrix viscoelasticity and viscoplasticity in mediating cell behaviors has only recently become appreciated, there is an abundance of opportunities for new fundamental knowledge in cell mechanics and key insights in applied areas such as wound healing (Fig. 2).^29,30^ While an elastic modulus has been reported for many soft tissues, the viscoelastic and viscoplastic properties of soft tissues at the microscale, the length-scale relevant to cell mechanotransduction, are unclear for many tissues. This characterization is critical to assessing the relevance of these findings to specific tissues and biological processes. In addition, while the mechanisms by which cells sense substrate elasticity in 2D culture are now well known, those mediating sensing of stress relaxation, particularly in 3D culture, remain unclear. Molecular clutch based-models have been successful at predicting cell responses to substrate elasticity and viscoelasticity in 2D culture, a context in which cells sense mechanics through integrin-based adhesions.^31^ However, in 3D culture, volume regulation and stretch activated channels have also been implicated in sensing matrix viscoelasticity.^32^ Elucidating the pathways by which cells sense matrix viscoelasticity, and how these interplay with the pathways that cells use to sense matrix stiffness, fibrillarity, and biochemical cues, will be an important task for the field in the coming decade (Fig. 2).

## IV. MORPHOGENESIS AND MICROPHYSIOLOGICAL SYSTEMS

Moving from the cellular level to higher order structures, tissue morphogenesis serves as a model system for engineering multi-cellular structures and organs-on-a-chip. Here, we describe embryonic development as an example of morphogenesis, along with the engineering of organ-on-a-chip systems.

### A. Cell and tissue biomechanics of morphogenesis

Embryonic development is a model of morphogenesis that can reveal fundamental knowledge of cell behavior in response to mechanical cues. For example, the mesendoderm of the *Xenopus* gastrula undergoes directed migration as a collective unit, but the cues that direct the spatiotemporal kinetics of migration are poorly understood. By dissociating the mesendoderm into single cells, the effect of cell–cell and cell–ECM interactions on cellular migration can be examined to reveal underlying signaling mechanisms, including the recruitment of keratin intermediate filaments at the rear and traction stresses at the front of the cell being driven by actomyosin pulling on integrins.^33^ In another model system, recent studies have shown that under suitable culture conditions, human pluripotent stem cells can undergo intricate morphogenetic events and self-organize to form patterned human embryo-like structures *in vitro.*^34–36^ These synthetic human embryonic tissues hold great promise for advancing our understanding of human embryology and reproductive medicine. For example, the effect of spatial patterning on neuroectoderm development can be studied in the pluripotent stem cell model of development, in which geometric confinement can be shown to mimic early neurulation by the regionalization of the neuroectoderm.^37^ However, there are still many aspects of *in vitro* culture systems that warrant improvement, including the determination of optimal chemical and mechanical properties of ECM to support embryonic growth, the presentation of microenvironmental factors in the niche, and the scale-up of the culture system for high-throughput screening of culture conditions or drugs.

The insights gained from understanding embryonic morphogenesis can be applied in the future for the treatment of congenital defects,^38^ which are a major cause of infant death.^39^ In particular, *in utero* stem cell therapy has the potential to revolutionize the treatment of congenital anomalies prior to birth. The fetal environment contains numerous qualities that may facilitate stem cell therapy, including the natural receptivity of the gestational environment to remodel and regenerate fetal tissues by stem cells.^40^ Recently, it has been shown that augmenting the *in utero* surgical repair of developmental defects with stem cells could functionally cure neural tube defects and associated motor function deficits at birth in large animal models.^41,42^

### B. Scalable mechanobiology

It is now widely appreciated that the mechanics, dimensionality, and other physical features of materials can strongly influence cell behavior. However, experimental platforms commonly used to probe these mechanobiological phenomena are challenging to reproducibly synthesize, labor-intensive, and/or difficult to deploy in combinatorial formats appropriate for screening. These characteristics limit the integration of mechanobiological concepts into the broader sphere of biology and medicine. Thus, the field desperately needs mechanobiological platforms that are scalable and parallelizable and can be integrated into standard pipelines for discovery, diagnosis, and screening. Early efforts to develop parallelized platforms for mechanobiology focused on retrofitting standard multi-well plate paradigms to accommodate engineered materials. This simple but powerful step greatly facilitated automated microscopy and drug screening.^43^ As the field has progressed, the tools of microfabrication and robotic spotting have been heavily leveraged to create microwell systems that allow combinatorial deployment of various material properties, including matrix stiffness, adhesivity, and enzymatic degradability.^44^

To reduce barriers to adoption, the new generation of platforms must also be sufficiently robust and user-friendly to promote use among biomedical and clinical scientists, even if this trades off to some degree against technological innovation/sophistication. Because many combinatorial mechanobiology systems require specialized equipment, such as microfabrication facilities or robotic spotters, there remains a need for platforms that can be fabricated using common laboratory equipment. For example, gradient photopatterning of hyaluronic acid hydrogels using orthogonal photochemistries and a simple UV light source has recently been used to fabricate two-dimensional arrays of matrix stiffness and adhesive ligand density on the same material.^45^ Enormous opportunity also exists to exploit organ-on-chip technologies for parallelized mechanobiology studies. This direction has been foreshadowed by the first lung-on-chip device, where mechanical stimulation of cells within the device strongly modulates responses to inflammatory stimuli.^46^ Finally, the incorporation of multiple cell types within a common platform remains a key challenge for the field. For example, in microscale tumor models, it is important to include not just the tumor cells but also associated stromal cells (e.g., fibroblasts, macrophages, and vasculature). There have been exciting first steps in these directions toward modeling glioblastoma (GBM) tumors using microfluidic strategies in which vessels are either allowed to self-assemble in 3D matrices^47^ or are represented as needle-molded channels.^48^ Three-dimensional printing has also recently emerged as a powerful strategy for integrating patient-derived tumor cells, vascular cells, and matrix.^49^ Another emerging application of scalable technology is the engineering of tissues with increasingly complex spatial geometries and cell types for regenerative medicine.^50^

### C. Organ-on-a-chip

Reproducing the human body *in vitro* is a dream that would allow having human samples available for drug testing, disease studies and corrections, and personalized medicine. By combining microfluidics with tissue engineering, numerous advances have been made in the organ-on-a-chip field to create small-scale, biological structures that recapitulate a specific organ function. Thus, these platforms have already been developed for the kidney, liver, heart, breast, gut, and blood vessels.^51^ Since they better mimic the hierarchical and physiological conditions seen *in vivo* than conventional cultures in dishes, they are especially attractive for assessing the toxicity of new drugs. For example, a human heart-liver-on-a-chip system has been developed for studying acetaminophen (APAP)-induced toxicity using primary human hepatocytes and induced pluripotent stem cell (iPSC)-derived cardiomyocytes [Fig. 1(d)].^52^

In the presence of APAP, liver toxicity could be functionally detected by a reduction of albumin production [Fig. 1(e)]. However, the combination of different modules with specific organs is required to reproduce the physiological complexity seen in the human body due to the interactions between different tissues and organs.^53^ If the microfluidics allow easy connections between different modules to build higher hierarchical systems, several challenges still remain before reaching the ultimate goal of a human body on chip. First, a specific organ should be engineered with human cells (rather than rodent cells). These cells may come from biopsies, or human stem cells and induced pluripotent stem cells can be used. Different methods for the differentiation of stem cells toward specific lineage have been established. Second, the structure, hierarchy, and functions of the organ should be reproduced. Currently, to approach the complexity of native organs, the technology uses organoid structures, which reproduce partially the functionalities of the targeted organs. Then, two or several organs that must be connected to obtain higher complexity in these inter-relations will influx on the functionality and responses of each organ. A common problem when culturing different tissues is to define a universal culture medium able to support the growth and maturation of different organs. One solution is the use of inner loops of perfusion with specialized culture media to feed each specific organ and a common outer loop of perfusion for connecting each organ together. The development of sensors is also needed to monitor each organ and their inter-relations. This analysis should be in real time and continuous. To this end, Khademhosseini's group has developed electrochemical sensors with regenerative capabilities.^54^

A goal of organ-on-a-chip technology is to reproduce *in vitro* specific structures of a tissue or organ to obtain optimal tissue functionalities that are similar to those seen *in vivo*. However, these full functionalities cannot be reached without inter-communication between organs. To support the growth and maturation of cells to obtain structural and hierarchical tissues, the combination of biomaterials and organ-on-a-chip may be advantageous. However, mimicking the complexity of the ECM and reproducing a cellular niche are challenging. Therefore, the development of biomaterials also aims toward more complexity and integration of several signals to cells. In the building of complex structures, the bioprinting technology has arisen due to its ability to deposit precisely cells and matrix in 3D. Apart from the printer technology itself, the development of new bioinks with adequate physical properties and good printability and supportive properties for the growth and differentiation of cells are important research areas. Currently, advancements in heterogeneous bioprinting with different cells and different materials are needed to enhance the complexity of the constructs obtained. Some attempts have been done on the use of multi-materials as bioinks with promising results.^55^ Additionally, the engineering of thick tissues requires the integration of vasculature for the delivery of oxygen, nutrients, and removal of waste. It is anticipated that the next generation of organ-on-a-chip platforms should integrate multi-materials, multi-cells, and vasculature to obtain tissues with enhanced complexity and hierarchical structures that are inter-related to each other, allowing a significant step toward the development of a human-on-a-chip system for disease modeling or drug screening applications (Fig. 2).^56^

## V. COMPUTATIONAL ANALYSIS OF CELLULAR NETWORKS

Computational simulations of biological processes have long been used to explore mechanistic models with quantitative rigor, at resolutions ranging from biochemical reactions to tissue-scale properties. In general, computational simulations are used to determine the broad plausibility of models or to test specific hypotheses by comparing simulated data with experimental data.^57^ For example, computationally tractable whole-cell models have been created of host–pathogen interactions from protein levels to cell–cell interactions.^58^ Such computational models can serve as a simulation tool for public access, use, and adaptation of other areas of research.^59^ Hybrid approaches that blend agent-based modeling with pharmacokinetic and pharmacodynamic modeling are powerful because they make simulations tractable while still retaining a grounding in physical mechanisms. For example, this approach has been used for studying tuberculosis to predict active vs latent infection and to explore the vast design space of antibiotic treatment.^60^

An intriguing application of computational simulations, especially multi-scale platforms that incorporate both intra-cellular regulatory networks and cell–cell interactions, is to use them to guide efforts to engineer complex multi-cellular phenomena such as morphogenesis [Fig. 1(f)].^2^ However, a major limitation of cell-based simulations to date is the lack of experimental data to constrain the initial models. With the advent of single cell molecular profiling technologies, such as mass cytometry,^61^ chromatin accessibility,^62^ and high throughput transcriptomics,^63–66^ the landscape is rapidly changing. For example, the Cahan laboratory recently developed a “cell typing” tool that determines the identity of a cell, as compared to a reference or annotated dataset, using single-cell RNA-Seq data. They further applied this approach to assess the fidelity of engineered cell populations, such as those derived from direct conversion or directed differentiation.^67^ One of the advances of this approach was that it is capable of performing cell typing even when the reference dataset was generated using different single cell RNA-Seq platforms or was from different species, opening up the prospect of leveraging rapidly accumulating sets of murine cell atlases to inform human single cell studies. Single-cell profiling can also be used to understand the molecular basis of how macrophages respond to environmental stimuli from a wide array of possible responses.^68^ One of the important advances of this work is the use of single-cell secretion profiling in conjunction with single-cell RNA-Seq, which links a critical functional readout to the molecular state of tumor-associated macrophages and their response to immunotherapy.

In the broader field of single-cell analytics, there are several areas that have received much attention and that we predict will feed into cell and molecular bioengineering. First, the existing algorithms can use single-cell RNA-Seq data to infer the position of each cell along a trajectory that represents progression along a biological process, such as differentiation or circadian rhythm.^69^ These trajectory inference algorithms are useful because they allow for the application of time-dependent analytical methods. For example, when applied to data from developmental stages, these methods can reveal regulators of cell fate decisions.^70^ Related to trajectory inference methods are algorithms to use the ratio of spliced-to-unspliced transcript abundance to predict the velocity or future transcriptional state of a cell.^71^ While the RNA velocity approaches go beyond inferring trajectories by determining directionality (for example, cells in cluster A are transitioning to cluster B), it is a very new technique that requires a deeper exploration, its limitations, and a fuller description of parameter customization. Third, there are computational methods to integrate *in situ* with single cell RNA-Seq data to infer the global transcriptional state and localization.^72,73^ One of the benefits of these methods is that they enable the inference of cell–cell interactions and help to characterize the influence of microanatomy on the expression state. While these methods can be informative, they are likely to be supplanted in the near future by better *in situ* sequencing technologies such as subcellular RNA-Seq, 3D intact-tissue single-cell sequencing, and spatially resolved single-cell sequencing.^74–77^ Finally, emerging methods can predict ligand–receptor interactions from single-cell RNA-Seq data. Most of these methods currently score putative interactions between clusters of cells based on known ligand–receptor interactions.^78–80^ We anticipate that in the near future more advanced computational techniques will yield more precise predictions by, for example, leveraging information about downstream signaling pathway targets. With the continual advancement of technologies that generate genome-wide data at a single-cell resolution, there are many opportunities for the development of clever algorithms that can help to optimally translate these big data into useful knowledge.

## VI. CONCLUSION

In the past few decades, the convergence of advanced technologies and computational biology has enabled a greater understanding of complex molecular, cellular, and extracellular interactions that regulate the tissue function. In this Perspectives piece, we have discussed the current state of the field, limitations in our understanding, and the opportunities ahead to develop more complex systems that better model tissue development, pathology, and regeneration. At the molecular level, IDPs and synthetic protein engineering can be applied toward applications such as imaging and modulation of cellular functions, and the addition of machine learning will further improve our fundamental understanding. At the multi-cellular level, organ-on-a-chip approaches in the future will incorporate multiple cell types, vascular networks, and more complex spatial geometries and bio-inks to better mimic physiological tissue complexity. Technological advances in computational modeling should enable more precise prediction of ligand–receptor interactions or inference of global transcriptional profiles from single cell RNA-Seq data. We anticipate that other future directions will include investigating the interactions of different cell types within complex multi-cellular systems at the single-cell resolution by using single cell RNA sequencing and *in situ* high throughput fluorescence *in situ* hybridization to map the cell phenotype and function. Other high-throughput technologies such as DNA microscopy may also provide new insights into the relationship among the DNA sequence, spatial organization, and cellular function. Additionally, the convergence of next generation sequencing with drug screening may enable the identification of new therapeutic treatments for a wide range of diseases.^81,82^ Despite the current challenges, we anticipate that molecular, cellular, and multi-cellular bioengineering approaches will become increasingly important in many aspects of biomedical research.

## References

[1] C. J. Oldfield and A. K. Dunker , “ Intrinsically disordered proteins and intrinsically disordered protein regions,” Annu. Rev. Biochem. 83, 553–584 (2014). 10.1146/annurev-biochem-072711-164947 24606139

[2] J. J. Velazquez , E. Su , P. Cahan , and M. R. Ebrahimkhani , “ Programming morphogenesis through systems and synthetic biology,” Trends Biotechnol. 36, 415–429 (2018). 10.1016/j.tibtech.2017.11.003 29229492 PMC6589336

[3] S. Milles , N. Salvi , M. Blackledge , and M. R. Jensen , “ Characterization of intrinsically disordered proteins and their dynamic complexes: From in vitro to cell-like environments,” Prog. Nucl. Magn. Reson. Spectrosc. 109, 79–100 (2018). 10.1016/j.pnmrs.2018.07.001 30527137

[4] D. Bracha , M. T. Walls , M. T. Wei , L. Zhu , M. Kurian , J. L. Avalos , J. E. Toettcher , and C. P. Brangwynne , “ Mapping local and global liquid phase behavior in living cells using photo-oligomerizable seeds,” Cell 175, 1467–1480.e1413 (2018). 10.1016/j.cell.2018.10.048 30500534 PMC6724719

[5] Y. Shin , Y. C. Chang , D. S. W. Lee , J. Berry , D. W. Sanders , P. Ronceray , N. S. Wingreen , M. Haataja , and C. P. Brangwynne , “ Liquid nuclear condensates mechanically sense and restructure the genome,” Cell 175, 1481–1491.e1413 (2018). 10.1016/j.cell.2018.10.057 30500535 PMC6724728

[6] K. M. Ruff , S. Roberts , A. Chilkoti , and R. V. Pappu , “ Advances in understanding stimulus-responsive phase behavior of intrinsically disordered protein polymers,” J. Mol. Biol. 430, 4619–4635 (2018). 10.1016/j.jmb.2018.06.031 29949750

[7] P. Limsakul , Q. Peng , Y. Wu , M. E. Allen , J. Liang , A. G. Remacle , T. Lopez , X. Ge , B. K. Kay , H. Zhao , A. Y. Strongin , X. L. Yang , S. Lu , and Y. Wang , “ Directed evolution to engineer monobody for FRET biosensor assembly and imaging at live-cell surface,” Cell Chem. Biol. 25, 370–379.e374 (2018). 10.1016/j.chembiol.2018.01.002 29396288 PMC5910193

[8] J. A. Seidel , A. Otsuka , and K. Kabashima , “ Anti-PD-1 and anti-CTLA-4 therapies in cancer: Mechanisms of action, efficacy, and limitations,” Front. Oncol. 8, 86 (2018). 10.3389/fonc.2018.00086 29644214 PMC5883082

[9] D. A. Silva , S. Yu , U. Y. Ulge , J. B. Spangler , K. M. Jude , C. Labao-Almeida , L. R. Ali , A. Quijano-Rubio , M. Ruterbusch , I. Leung , T. Biary , S. J. Crowley , E. Marcos , C. D. Walkey , B. D. Weitzner , F. Pardo-Avila , J. Castellanos , L. Carter , L. Stewart , S. R. Riddell , M. Pepper , G. J. L. Bernardes , M. Dougan , K. C. Garcia , and D. Baker , “ De novo design of potent and selective mimics of IL-2 and IL-15,” Nature 565, 186–191 (2019). 10.1038/s41586-018-0830-7 30626941 PMC6521699

[10] B. S. Schuster , E. H. Reed , R. Parthasarathy , C. N. Jahnke , R. M. Caldwell , J. G. Bermudez , H. Ramage , M. C. Good , and D. A. Hammer , “ Controllable protein phase separation and modular recruitment to form responsive membraneless organelles,” Nat. Commun. 9, 2985 (2018). 10.1038/s41467-018-05403-1 30061688 PMC6065366

[11] J. L. Neira , J. Bintz , M. Arruebo , B. Rizzuti , T. Bonacci , S. Vega , A. Lanas , A. Velazquez-Campoy , J. L. Iovanna , and O. Abian , “ Identification of a drug targeting an intrinsically disordered protein involved in pancreatic adenocarcinoma,” Sci. Rep. 7, 39732 (2017). 10.1038/srep39732 28054562 PMC5213423

[12] B. Koepnick , J. Flatten , T. Husain , A. Ford , D. A. Silva , M. J. Bick , A. Bauer , G. Liu , Y. Ishida , A. Boykov , R. D. Estep , S. Kleinfelter , T. Norgard-Solano , L. Wei , F. Players , G. T. Montelione , F. DiMaio , Z. Popovic , F. Khatib , S. Cooper , and D. Baker , “ De novo protein design by citizen scientists,” Nature 570, 390–394 (2019). 10.1038/s41586-019-1274-4 31168091 PMC6701466

[13] K. Kretzschmar and H. Clevers , “ Organoids: Modeling development and the stem cell niche in a dish,” Dev. Cell 38, 590–600 (2016). 10.1016/j.devcel.2016.08.014 27676432

[14] M. J. Dalby , N. Gadegaard , R. Tare , A. Andar , M. O. Riehle , P. Herzyk , C. D. Wilkinson , and R. O. Oreffo , “ The control of human mesenchymal cell differentiation using nanoscale symmetry and disorder,” Nat. Mater. 6, 997–1003 (2007). 10.1038/nmat2013 17891143

[15] A. J. Engler , S. Sen , H. L. Sweeney , and D. E. Discher , “ Matrix elasticity directs stem cell lineage specification,” Cell 126, 677–689 (2006). 10.1016/j.cell.2006.06.044 16923388

[16] Q. L. Loh and C. Choong , “ Three-dimensional scaffolds for tissue engineering applications: Role of porosity and pore size,” Tissue Eng. Part B: Rev. 19, 485–502 (2013). 10.1089/ten.teb.2012.0437 23672709 PMC3826579

[17] S. Khetan , M. Guvendiren , W. R. Legant , D. M. Cohen , C. S. Chen , and J. A. Burdick , “ Degradation-mediated cellular traction directs stem cell fate in covalently crosslinked three-dimensional hydrogels,” Nat. Mater. 12, 458–465 (2013). 10.1038/nmat3586 23524375 PMC3633615

[18] C. J. Flaim , S. Chien , and S. N. Bhatia , “ An extracellular matrix microarray for probing cellular differentiation,” Nat. Methods 2, 119–125 (2005). 10.1038/nmeth736 15782209

[19] M. Guvendiren and J. A. Burdick , “ Stiffening hydrogels to probe short- and long-term cellular responses to dynamic mechanics,” Nat. Commun. 3, 792 (2012). 10.1038/ncomms1792 22531177

[20] S. Nasrollahi , C. Walter , A. J. Loza , G. V. Schimizzi , G. D. Longmore , and A. Pathak , “ Past matrix stiffness primes epithelial cells and regulates their future collective migration through a mechanical memory,” Biomaterials 146, 146–155 (2017). 10.1016/j.biomaterials.2017.09.012 28918264 PMC5659718

[21] M. Guvendiren and J. A. Burdick , “ Stem cell response to spatially and temporally displayed and reversible surface topography,” Adv. Healthcare Mater. 2, 155–164 (2013). 10.1002/adhm.201200105 23184470

[22] C. Yang , F. W. DelRio , H. Ma , A. R. Killaars , L. P. Basta , K. A. Kyburz , and K. S. Anseth , “ Spatially patterned matrix elasticity directs stem cell fate,” Proc. Natl. Acad. Sci. U. S. A. 113, E4439–E4445 (2016). 10.1073/pnas.1609731113 27436901 PMC4978284

[23] O. Chaudhuri , S. T. Koshy , C. Branco da Cunha , J. W. Shin , C. S. Verbeke , K. H. Allison , and D. J. Mooney , “ Extracellular matrix stiffness and composition jointly regulate the induction of malignant phenotypes in mammary epithelium,” Nat. Mater. 13, 970–978 (2014). 10.1038/nmat4009 24930031

[24] O. Chaudhuri , L. Gu , D. Klumpers , M. Darnell , S. A. Bencherif , J. C. Weaver , N. Huebsch , H. P. Lee , E. Lippens , G. N. Duda , and D. J. Mooney , “ Hydrogels with tunable stress relaxation regulate stem cell fate and activity,” Nat. Mater. 15, 326–334 (2016). 10.1038/nmat4489 26618884 PMC4767627

[25] A. R. Cameron , J. E. Frith , and J. J. Cooper-White , “ The influence of substrate creep on mesenchymal stem cell behaviour and phenotype,” Biomaterials 32, 5979–5993 (2011). 10.1016/j.biomaterials.2011.04.003 21621838

[26] D. D. McKinnon , D. W. Domaille , J. N. Cha , and K. S. Anseth , “ Biophysically defined and cytocompatible covalently adaptable networks as viscoelastic 3D cell culture systems,” Adv. Mater. 26, 865–872 (2014). 10.1002/adma.201303680 24127293 PMC4582033

[27] H. P. Lee , L. Gu , D. J. Mooney , M. E. Levenston , and O. Chaudhuri , “ Mechanical confinement regulates cartilage matrix formation by chondrocytes,” Nat. Mater. 16, 1243–1251 (2017). 10.1038/nmat4993 28967913 PMC5701824

[28] K. M. Wisdom , K. Adebowale , J. Chang , J. Y. Lee , S. Nam , R. Desai , N. S. Rossen , M. Rafat , R. B. West , L. Hodgson , and O. Chaudhuri , “ Matrix mechanical plasticity regulates cancer cell migration through confining microenvironments,” Nat. Commun. 9, 4144 (2018). 10.1038/s41467-018-06641-z 30297715 PMC6175826

[29] A. S. Liu , H. Wang , C. R. Copeland , C. S. Chen , V. B. Shenoy , and D. H. Reich , “ Matrix viscoplasticity and its shielding by active mechanics in microtissue models: Experiments and mathematical modeling,” Sci. Rep. 6, 33919 (2016). 10.1038/srep33919 27671239 PMC5037370

[30] S. J. Dubois , N. Kalashnikov , and C. Moraes , “ Robust and precise wounding and analysis of engineered contractile tissues,” Tissue Eng., Part C: Methods 25, 677–686 (2019). 10.1089/ten.tec.2019.0123 31411125

[31] Z. Gong , S. E. Szczesny , S. R. Caliari , E. E. Charrier , O. Chaudhuri , X. Cao , Y. Lin , R. L. Mauck , P. A. Janmey , J. A. Burdick , and V. B. Shenoy , “ Matching material and cellular timescales maximizes cell spreading on viscoelastic substrates,” Proc. Natl. Acad. Sci. U. S. A. 115, E2686–E2695 (2018). 10.1073/pnas.1716620115 29507238 PMC5866566

[32] H. P. Lee , R. Stowers , and O. Chaudhuri , “ Volume expansion and TRPV4 activation regulate stem cell fate in three-dimensional microenvironments,” Nat. Commun. 10, 529 (2019). 10.1038/s41467-019-08465-x 30705265 PMC6355972

[33] P. Sonavane , C. Wang , B. Dzamba , D. Shook , and D. DeSimone , “ Coordination of collective cell movements at gastrulation is responsive to changes in mechanical environment (abstract),” in Cell and Molecular Bioengineering Conference, Coronado, CA, USA (2019).

[34] Y. Shao , K. Taniguchi , K. Gurdziel , R. F. Townshend , X. Xue , K. M. A. Yong , J. Sang , J. R. Spence , D. L. Gumucio , and J. Fu , “ Self-organized amniogenesis by human pluripotent stem cells in a biomimetic implantation-like niche,” Nat. Mater. 16, 419–425 (2017). 10.1038/nmat4829 27941807 PMC5374007

[35] Y. Shao , K. Taniguchi , R. F. Townshend , T. Miki , D. L. Gumucio , and J. Fu , “ A pluripotent stem cell-based model for post-implantation human amniotic sac development,” Nat. Commun. 8, 208 (2017). 10.1038/s41467-017-00236-w 28785084 PMC5547056

[36] X. Xue , Y. Sun , A. M. Resto-Irizarry , Y. Yuan , K. M. Aw Yong , Y. Zheng , S. Weng , Y. Shao , Y. Chai , L. Studer , and J. Fu , “ Mechanics-guided embryonic patterning of neuroectoderm tissue from human pluripotent stem cells,” Nat. Mater. 17, 633–641 (2018). 10.1038/s41563-018-0082-9 29784997 PMC6622450

[37] J. Fu , “ Synthetic human embryo-like structures: A new paradigm for human embryology (abstract),” in Cell and Molecular Bioengineering Conference, Coronado, CA, USA (2019).

[38] See http://ephtracking.cdc.gov/showBirthDefects.action for “CDC, Birth Defects, 2012.”

[39] J. A. Martin , K. D. Kochanek , D. M. Strobino , B. Guyer , and M. F. MacDorman , “ Annual summary of vital statistics—2003,” Pediatrics 115, 619–634 (2005). 10.1542/peds.2004-2695 15741364

[40] E. Tiblad and M. Westgren , “ Fetal stem-cell transplantation,” Best Pract. Res. Clin. Obstet. Gynaecol. 22, 189–201 (2008). 10.1016/j.bpobgyn.2007.07.007 18035592

[41] S. Kabagambe , B. Keller , J. Becker , L. Goodman , C. Pivetti , L. Lankford , K. Chung , C. Lee , Y. J. Chen , P. Kumar , M. Vanover , A. Wang , and D. Farmer , “ Placental mesenchymal stromal cells seeded on clinical grade extracellular matrix improve ambulation in ovine myelomeningocele,” J. Pediatr Surg. 53, 178–182 (2018). 10.1016/j.jpedsurg.2017.10.032 29122293

[42] A. Wang , E. G. Brown , L. Lankford , B. A. Keller , C. D. Pivetti , N. A. Sitkin , M. S. Beattie , J. C. Bresnahan , and D. L. Farmer , “ Placental mesenchymal stromal cells rescue ambulation in ovine myelomeningocele,” Stem Cells Transl. Med. 4, 659–669 (2015). 10.5966/sctm.2014-0296 25911465 PMC4449103

[43] J. D. Mih , A. S. Sharif , F. Liu , A. Marinkovic , M. M. Symer , and D. J. Tschumperlin , “ A multiwell platform for studying stiffness-dependent cell biology,” PLoS One 6, e19929 (2011). 10.1371/journal.pone.0019929 21637769 PMC3103526

[44] S. Gobaa , S. Hoehnel , M. Roccio , A. Negro , S. Kobel , and M. P. Lutolf , “ Artificial niche microarrays for probing single stem cell fate in high throughput,” Nat. Methods 8, 949–955 (2011). 10.1038/nmeth.1732 21983923

[45] A. D. Rape , M. Zibinsky , N. Murthy , and S. Kumar , “ A synthetic hydrogel for the high-throughput study of cell-ECM interactions,” Nat. Commun. 6, 8129 (2015). 10.1038/ncomms9129 26350361 PMC4566157

[46] D. Huh , B. D. Matthews , A. Mammoto , M. Montoya-Zavala , H. Y. Hsin , and D. E. Ingber , “ Reconstituting organ-level lung functions on a chip,” Science 328, 1662–1668 (2010). 10.1126/science.1188302 20576885 PMC8335790

[47] Y. Xiao , D. Kim , B. Dura , K. Zhang , R. Yan , H. Li , E. Han , J. Ip , P. Zou , J. Liu , A. T. Chen , A. O. Vortmeyer , J. Zhou , and R. Fan , “ Ex vivo dynamics of human glioblastoma cells in a microvasculature-on-a-chip system correlates with tumor heterogeneity and subtypes,” Adv. Sci. 6, 1801531 (2019). 10.1002/advs.201801531 PMC6468969 31016107

[48] K. J. Wolf , S. Lee , and S. Kumar , “ A 3D topographical model of parenchymal infiltration and perivascular invasion in glioblastoma,” APL Bioeng. 2, 031903 (2018). 10.1063/1.5021059 29855630 PMC5971843

[49] H. G. Yi , Y. H. Jeong , Y. Kim , Y. J. Choi , H. E. Moon , S. H. Park , K. S. Kang , M. Bae , J. Jang , H. Youn , S. H. Paek , and D. W. Cho , “ A bioprinted human-glioblastoma-on-a-chip for the identification of patient-specific responses to chemoradiotherapy,” Nat. Biomed. Eng. 3, 509–519 (2019). 10.1038/s41551-019-0363-x 31148598

[50] B. Grigoryan , S. J. Paulsen , D. C. Corbett , D. W. Sazer , C. L. Fortin , A. J. Zaita , P. T. Greenfield , N. J. Calafat , J. P. Gounley , A. H. Ta , F. Johansson , A. Randles , J. E. Rosenkrantz , J. D. Louis-Rosenberg , P. A. Galie , K. R. Stevens , and J. S. Miller , “ Multivascular networks and functional intravascular topologies within biocompatible hydrogels,” Science 364, 458–464 (2019). 10.1126/science.aav9750 31048486 PMC7769170

[51] S. Selimovic , M. R. Dokmeci , and A. Khademhosseini , “ Organs-on-a-chip for drug discovery,” Curr. Opin. Pharmacol. 13, 829–833 (2013). 10.1016/j.coph.2013.06.005 23850526

[52] N. S. Bhise , V. Manoharan , S. Massa , A. Tamayol , M. Ghaderi , M. Miscuglio , Q. Lang , Y. S. Zhang , S. R. Shin , G. Calzone , N. Annabi , T. D. Shupe , C. E. Bishop , A. Atala , M. R. Dokmeci , and A. Khademhosseini , “ A liver-on-a-chip platform with bioprinted hepatic spheroids,” Biofabrication 8, 014101 (2016). 10.1088/1758-5090/8/1/014101 26756674

[53] A. Skardal , S. V. Murphy , M. Devarasetty , I. Mead , H. W. Kang , Y. J. Seol , Y. S. Zhang , S. R. Shin , L. Zhao , J. Aleman , A. R. Hall , T. D. Shupe , A. Kleensang , M. R. Dokmeci , S. Jin Lee , J. D. Jackson , J. J. Yoo , T. Hartung , A. Khademhosseini , S. Soker , C. E. Bishop , and A. Atala , “ Multi-tissue interactions in an integrated three-tissue organ-on-a-chip platform,” Sci. Rep. 7, 8837 (2017). 10.1038/s41598-017-08879-x 28821762 PMC5562747

[54] Y. S. Zhang , J. Aleman , S. R. Shin , T. Kilic , D. Kim , S. A. Mousavi Shaegh , S. Massa , R. Riahi , S. Chae , N. Hu , H. Avci , W. Zhang , A. Silvestri , A. Sanati Nezhad , A. Manbohi , F. De Ferrari , A. Polini , G. Calzone , N. Shaikh , P. Alerasool , E. Budina , J. Kang , N. Bhise , J. Ribas , A. Pourmand , A. Skardal , T. Shupe , C. E. Bishop , M. R. Dokmeci , A. Atala , and A. Khademhosseini , “ Multisensor-integrated organs-on-chips platform for automated and continual in situ monitoring of organoid behaviors,” Proc. Natl. Acad. Sci. U. S. A. 114, E2293–E2302 (2017). 10.1073/pnas.1612906114 28265064 PMC5373350

[55] W. Liu , Y. S. Zhang , M. A. Heinrich , F. De Ferrari , H. L. Jang , S. M. Bakht , M. M. Alvarez , J. Yang , Y. C. Li , G. Trujillo-de Santiago , A. K. Miri , K. Zhu , P. Khoshakhlagh , G. Prakash , H. Cheng , X. Guan , Z. Zhong , J. Ju , G. H. Zhu , X. Jin , S. R. Shin , M. R. Dokmeci , and A. Khademhosseini , “ Rapid continuous multimaterial extrusion bioprinting,” Adv. Mater. 29, 1604630 (2017). 10.1002/adma.201604630 PMC5235978 27859710

[56] S. Jalili-Firoozinezhad , F. S. Gazzaniga , E. L. Calamari , D. M. Camacho , C. W. Fadel , A. Bein , B. Swenor , B. Nestor , M. J. Cronce , A. Tovaglieri , O. Levy , K. E. Gregory , D. T. Breault , J. M. S. Cabral , D. L. Kasper , R. Novak , and D. E. Ingber , “ A complex human gut microbiome cultured in an anaerobic intestine-on-a-chip,” Nat. Biomed. Eng. 3, 520–531 (2019). 10.1038/s41551-019-0397-0 31086325 PMC6658209

[57] J. Sharpe , “ Computer modeling in developmental biology: Growing today, essential tomorrow,” Development 144, 4214–4225 (2017). 10.1242/dev.151274 29183935

[58] M. Covert , “ A multi-scale, integrated approach to understanding infection (abstract),” in Cell and Molecular Bioengineering Conference, Coronado, CA, USA (2019).

[59] R. Lee , J. R. Karr , and M. W. Covert , “ WholeCellViz: Data visualization for whole-cell models,” BMC Bioinf. 14, 253 (2013). 10.1186/1471-2105-14-253 PMC3765349 23964998

[60] E. Pienaar , J. Sarathy , B. Prideaux , J. Dietzold , V. Dartois , D. E. Kirschner , and J. J. Linderman , “ Comparing efficacies of moxifloxacin, levofloxacin and gatifloxacin in tuberculosis granulomas using a multi-scale systems pharmacology approach,” PLoS Comput. Biol. 13, e1005650 (2017). 10.1371/journal.pcbi.1005650 28817561 PMC5560534

[61] S. C. Bendall , E. F. Simonds , P. Qiu , A. D. Amir el , P. O. Krutzik , R. Finck , R. V. Bruggner , R. Melamed , A. Trejo , O. I. Ornatsky , R. S. Balderas , S. K. Plevritis , K. Sachs , D. Pe'er , S. D. Tanner , and G. P. Nolan , “ Single-cell mass cytometry of differential immune and drug responses across a human hematopoietic continuum,” Science 332, 687–696 (2011). 10.1126/science.1198704 21551058 PMC3273988

[62] J. D. Buenrostro , B. Wu , U. M. Litzenburger , D. Ruff , M. L. Gonzales , M. P. Snyder , H. Y. Chang , and W. J. Greenleaf , “ Single-cell chromatin accessibility reveals principles of regulatory variation,” Nature 523, 486–490 (2015). 10.1038/nature14590 26083756 PMC4685948

[63] A. M. Klein , L. Mazutis , I. Akartuna , N. Tallapragada , A. Veres , V. Li , L. Peshkin , D. A. Weitz , and M. W. Kirschner , “ Droplet barcoding for single-cell transcriptomics applied to embryonic stem cells,” Cell 161, 1187–1201 (2015). 10.1016/j.cell.2015.04.044 26000487 PMC4441768

[64] E. Z. Macosko , A. Basu , R. Satija , J. Nemesh , K. Shekhar , M. Goldman , I. Tirosh , A. R. Bialas , N. Kamitaki , E. M. Martersteck , J. J. Trombetta , D. A. Weitz , J. R. Sanes , A. K. Shalek , A. Regev , and S. A. McCarroll , “ Highly parallel genome-wide expression profiling of individual cells using nanoliter droplets,” Cell 161, 1202–1214 (2015). 10.1016/j.cell.2015.05.002 26000488 PMC4481139

[65] G. X. Zheng , J. M. Terry , P. Belgrader , P. Ryvkin , Z. W. Bent , R. Wilson , S. B. Ziraldo , T. D. Wheeler , G. P. McDermott , J. Zhu , M. T. Gregory , J. Shuga , L. Montesclaros , J. G. Underwood , D. A. Masquelier , S. Y. Nishimura , M. Schnall-Levin , P. W. Wyatt , C. M. Hindson , R. Bharadwaj , A. Wong , K. D. Ness , L. W. Beppu , H. J. Deeg , C. McFarland , K. R. Loeb , W. J. Valente , N. G. Ericson , E. A. Stevens , J. P. Radich , T. S. Mikkelsen , B. J. Hindson , and J. H. Bielas , “ Massively parallel digital transcriptional profiling of single cells,” Nat. Commun. 8, 14049 (2017). 10.1038/ncomms14049 28091601 PMC5241818

[66] C. S. McGinnis , D. M. Patterson , J. Winkler , D. N. Conrad , M. Y. Hein , V. Srivastava , J. L. Hu , L. M. Murrow , J. S. Weissman , Z. Werb , E. D. Chow , and Z. J. Gartner , “ MULTI-seq: Sample multiplexing for single-cell RNA sequencing using lipid-tagged indices,” Nat. Methods 16, 619–626 (2019). 10.1038/s41592-019-0433-8 31209384 PMC6837808

[67] Y. Tan and P. Cahan , “ SingleCellNet: A computational tool to classify single cell RNA-Seq data across platforms and across species,” Cell Syst. 9, 207–213.e202 (2019). 10.1016/j.cels.2019.06.004 31377170 PMC6715530

[68] K. Miller-Jensen , “ Dissecting macrophage regulation and functions with single-cell secretion profiling,” in Cell and Molecular Bioengineering Conference, Coronado, CA, USA (2019).

[69] W. Saelens , R. Cannoodt , H. Todorov , and Y. Saeys , “ A comparison of single-cell trajectory inference methods,” Nat. Biotechnol. 37, 547–554 (2019). 10.1038/s41587-019-0071-9 30936559

[70] C. Trapnell , D. Cacchiarelli , J. Grimsby , P. Pokharel , S. Li , M. Morse , N. J. Lennon , K. J. Livak , T. S. Mikkelsen , and J. L. Rinn , “ The dynamics and regulators of cell fate decisions are revealed by pseudotemporal ordering of single cells,” Nat. Biotechnol. 32, 381–386 (2014). 10.1038/nbt.2859 24658644 PMC4122333

[71] G. L. Manno , R. Soldatov , A. Zeisel , E. Braun , H. Hochgerner , V. Petukhov , K. Lidschreiber , M. E. Kastriti , P. Lonnerberg , A. Furlan , J. Fan , L. E. Borm , Z. Liu , D. van Bruggen , J. Guo , X. He , R. Barker , E. Sundstrom , G. Castelo-Branco , P. Cramer , I. Adameyko , S. Linnarsson , and P. V. Kharchenko , “ RNA velocity of single cells,” Nature 560, 494–498 (2018). 10.1038/s41586-018-0414-6 30089906 PMC6130801

[72] K. Achim , J. B. Pettit , L. R. Saraiva , D. Gavriouchkina , T. Larsson , D. Arendt , and J. C. Marioni , “ High-throughput spatial mapping of single-cell RNA-seq data to tissue of origin,” Nat. Biotechnol. 33, 503–509 (2015). 10.1038/nbt.3209 25867922

[73] R. Satija , J. A. Farrell , D. Gennert , A. F. Schier , and A. Regev , “ Spatial reconstruction of single-cell gene expression data,” Nat. Biotechnol. 33, 495–502 (2015). 10.1038/nbt.3192 25867923 PMC4430369

[74] J. H. Lee , E. R. Daugharthy , J. Scheiman , R. Kalhor , J. L. Yang , T. C. Ferrante , R. Terry , S. S. Jeanty , C. Li , R. Amamoto , D. T. Peters , B. M. Turczyk , A. H. Marblestone , S. A. Inverso , A. Bernard , P. Mali , X. Rios , J. Aach , and G. M. Church , “ Highly multiplexed subcellular RNA sequencing in situ,” Science 343, 1360–1363 (2014). 10.1126/science.1250212 24578530 PMC4140943

[75] X. Wang , W. E. Allen , M. A. Wright , E. L. Sylwestrak , N. Samusik , S. Vesuna , K. Evans , C. Liu , C. Ramakrishnan , J. Liu , G. P. Nolan , F. A. Bava , and K. Deisseroth , “ Three-dimensional intact-tissue sequencing of single-cell transcriptional states,” Science 361, eaat5691 (2018). 10.1126/science.aat5691 29930089 PMC6339868

[76] K. H. Chen , A. N. Boettiger , J. R. Moffitt , S. Wang , and X. Zhuang , “ RNA imaging. Spatially resolved, highly multiplexed RNA profiling in single cells,” Science 348, aaa6090 (2015). 10.1126/science.aaa6090 25858977 PMC4662681

[77] S. G. Rodriques , R. R. Stickels , A. Goeva , C. A. Martin , E. Murray , C. R. Vanderburg , J. Welch , L. M. Chen , F. Chen , and E. Z. Macosko , “ Slide-seq: A scalable technology for measuring genome-wide expression at high spatial resolution,” Science 363, 1463–1467 (2019). 10.1126/science.aaw1219 30923225 PMC6927209

[78] R. Menon , E. A. Otto , A. Kokoruda , J. Zhou , Z. Zhang , E. Yoon , Y. C. Chen , O. Troyanskaya , J. R. Spence , M. Kretzler , and C. Cebrian , “ Single-cell analysis of progenitor cell dynamics and lineage specification in the human fetal kidney,” Development 145, dev164038 (2018). 10.1242/dev.164038 30166318 PMC6124540

[79] M. P. Kumar , J. Du , G. Lagoudas , Y. Jiao , A. Sawyer , D. C. Drummond , D. A. Lauffenburger , and A. Raue , “ Analysis of single-cell RNA-Seq identifies cell-cell communication associated with tumor characteristics,” Cell Rep. 25, 1458–1468.e1454 (2018). 10.1016/j.celrep.2018.10.047 30404002 PMC7009724

[80] D. A. Skelly , G. T. Squiers , M. A. McLellan , M. T. Bolisetty , P. Robson , N. A. Rosenthal , and A. R. Pinto , “ Single-cell transcriptional profiling reveals cellular diversity and intercommunication in the mouse heart,” Cell Rep. 22, 600–610 (2018). 10.1016/j.celrep.2017.12.072 29346760

[81] S. W. Song , S. D. Kim , D. Y. Oh , Y. Lee , A. C. Lee , Y. Jeong , H. J. Bae , D. Lee , S. Lee , J. Kim , and S. Kwon , “ One-step generation of a drug-releasing hydrogel microarray-on-a-chip for large-scale sequential drug combination screening,” Adv. Sci. 6, 1801380 (2019). 10.1002/advs.201801380 PMC6364496 30775230

[82] A. C. Lee , Y. Lee , D. Lee , and S. Kwon , “ Divide and conquer: A perspective on biochips for single-cell and rare-molecule analysis by next-generation sequencing,” APL Bioeng. 3, 020901 (2019). 10.1063/1.5095962 31431936 PMC6697027

